# Crystal structure of η″-Fe_3_Al_7+*x*_ determined by single-crystal synchrotron X-ray diffraction combined with scanning transmission electron microscopy

**DOI:** 10.1080/14686996.2019.1613174

**Published:** 2019-06-06

**Authors:** Norihiko L. Okamoto, Masaya Higashi, Haruyuki Inui

**Affiliations:** a Department of Materials Science and Engineering, Kyoto University, Kyoto, Japan; b Center for Elements Strategy Initiative for Structure Materials (ESISM), Kyoto University, Kyoto, Japan; c Institute for Materials Research, Tohoku University, Sendai, Japan

**Keywords:** Intermetallic compound, superlattice structure, orientation variants, nanoscale twins, icosahedron, 10 Engineering and Structural materials, 106 Metallic materials, 212 Surface and interfaces, 302 Crystallization / Heat treatment / Crystal growth, 503 TEM, STEM, SEM, 504 X-ray / Neutron diffraction and scattering

## Abstract

The crystal structure of η″-Fe_3_Al_7+*x*_, the low-temperature phase of η-Fe_2_Al_5_ with a composition on the Fe-rich side of the solid solubility range, has been determined by synchrotron X-ray single-crystal diffraction combined with scanning transmission electron microscopy. The η″ phase possesses commensurate long-period-ordered superlattice structures (space group *Pmcn*) based on the parent orthorhombic unit cell of η-Fe_2_Al_5_, consisting of twin domains (orientation variants) alternately stacked along the long-periodicity axis. Each of the twin domains possesses a motif structure belonging to the base-centered monoclinic space group *C*2/*m*, with a cell volume twice that of the parent orthorhombic unit cell (space group *Cmcm*). One-fourth of the *c*-axis chain sites corresponding to Al2- and Al3-sites in the η phase are respectively occupied by both Fe and Al atoms and exclusively by Al atoms in a regular manner. This regularity is disturbed in the twin-boundary region, giving rise to structural/compositional modulation. Because of the different chemical compositions between the motif structure and twin-boundary region, the η″ phase with various compositions can be constructed only by changing the number of the parent orthorhombic unit cells to be stacked along the orthorhombic *c*-axis, without changing the atomic arrangements for the motif structure or the twin boundary to account for the observed solid solubility range. The chemical formula of the η″ phase can thus be expressed as Fe_3_Al_7+*x*_ under a simple assumption on the occupancies for Al/Fe atoms in the *c*-axis chain sites.

## Graphical Abstract

**Figure:**
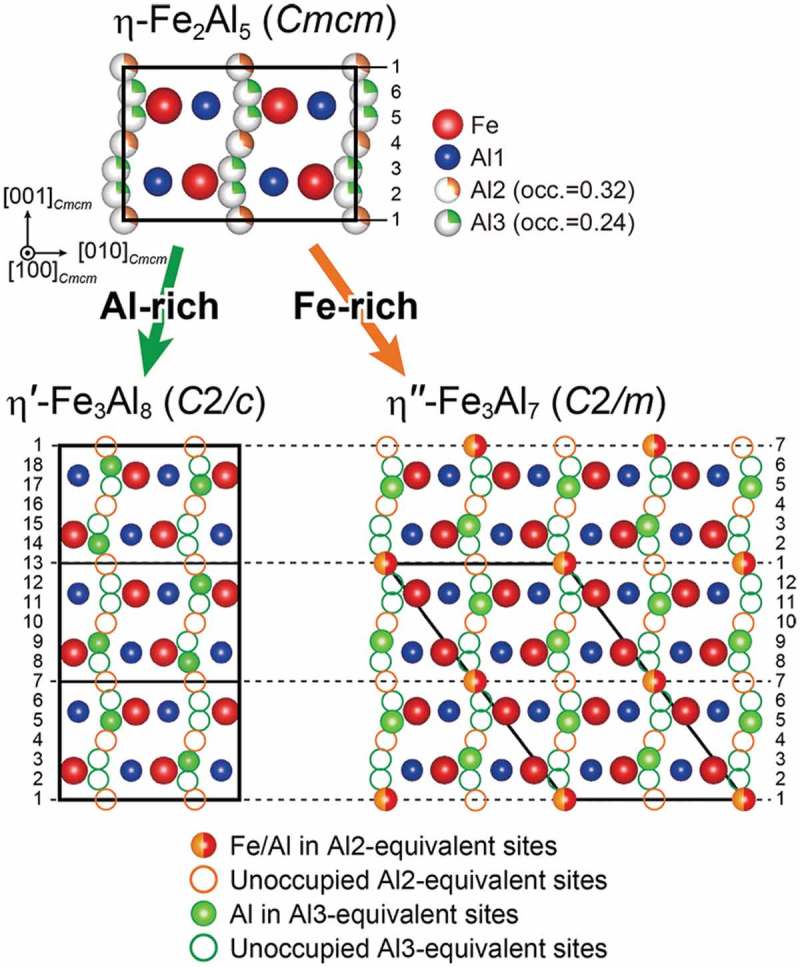

## 1. Introduction

Aluminized steels have received increasing attention in recent years, because of their excellent resistance to corrosion and oxidation at high temperatures as well as high optical reflectivity, leading to good design properties. They have been widely used in fields such as automobile exhaust systems, household appliances, and building materials in severe environments that Zn-coated steels cannot tolerate [1–6]. The global depletion of Zn resource has also driven the transition from Zn- to Al-coated steels [7,8]. Hot-dip aluminized steels are produced by immersing steel strips into a molten Al or Al-Si bath, followed by heat treatment to alloy the Al coating over the Fe substrate through thermal diffusion. A thin aluminum oxide layer formed on top of the coating is believed to act as a diffusion and thermal barrier, enhancing the corrosion and thermal resistance [2,3]. Below the aluminum oxide layer, the brittle intermetallic compound η-Fe_2_Al_5_ forms as the major phase in the reaction layer, especially when the molten bath is pure Al, exhibiting a columnar texture elongated along its orthorhombic [001] direction [9–11]. Therefore, the η-Fe_2_Al_5_ phase is expected to play a decisive role in determining the mechanical and other properties of hot-dip aluminized steels [12,13]. Furthermore, the η-Fe_2_Al_5_ phase is also known to act as an inhibition layer during the galvanizing/galvannealing process to suppress the excessively rapid reaction of the Zn coating with the Fe substrate [14]. Thus, understanding the structural [11,15,16], thermodynamic [17–21], and mechanical [9,12,13] properties of η-Fe_2_Al_5_ is indispensable for the development of Al- and Zn-coated steels.

**Figure 1.**
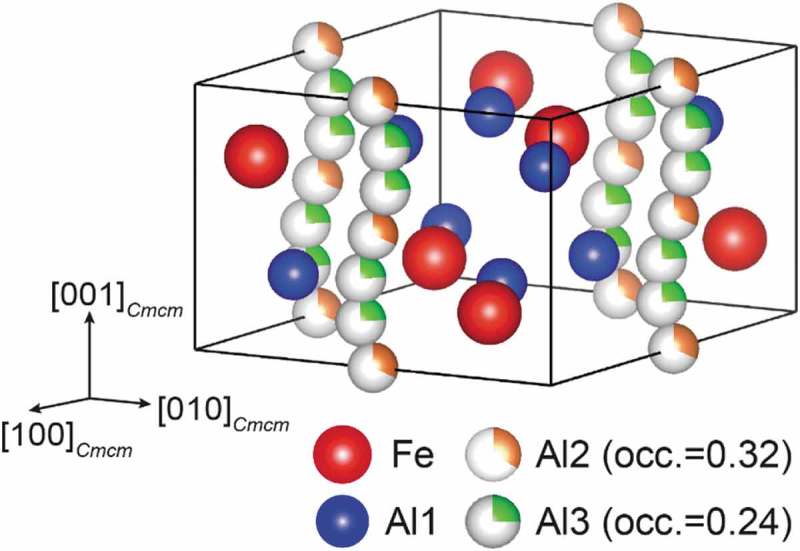
Crystal structure of the η-Fe_2_Al_5_ phase reported by Burkhardt et al. [15]. The six Al sites with partial occupancies (Al2 and Al3) are arranged along the *c*-axis of the orthorhombic unit cell.

According to Burkhardt et al. [15], the crystal structure of η-Fe_2_Al_5_ belongs to the orthorhombic space group *Cmcm*, consisting of an FeAl_2_ framework structure (Fe and Al1 sites with full occupancies) and a chain of six Al sites with partial occupancies (0.32 and 0.24 for Al2 and Al3 sites, respectively) along the orthorhombic *c*-axis (Figure 1). The relatively wide solid solubility range (Al-27 to 31 at.% Fe) reported for the η phase [19–21] is usually assumed to be realized by varying Al occupancies in the *c*-axis chain sites. However, Becker et al. [22,23] have recently reported that not only Al but also Fe atoms occupy the *c*-axis chain sites in the η phase with Fe-rich compositions, suggesting that the number density of atoms in the *c*-axis chain does not depend much on the alloy composition in the solubility range, unlike the expectation from the crystal structure model by Burkhardt et al. [15]. Very recently, a few research groups including ours have independently found that there are several different low-temperature phases having crystal structures based on the η phase, with the Al/Fe atoms arranged in ordered manners in the orthorhombic *c*-axis chain [22–24]. On the Al-rich side of the solubility range (~Al-27.3 at.%Fe), Al atoms in the *c*-axis chain are arranged in an ordered manner to form a new phase (η′-Fe_3_Al_8_) with a tripled superlattice structure based on the parent η structure [24]. We have found that in the η′ phase, one-third of the *c*-axis chain sites corresponding to the Al3 sites in the η phase are occupied exclusively by Al atoms, while those corresponding to the Al2 sites are completely vacant. As a result, a total of two-ninths of the *c*-axis chain sites are occupied in an ordered manner [24]. Becker et al. [22,23] have reported, on the other hand, that when the alloy composition is on the Fe-rich side of the solubility range (~Al-29.4 at.%Fe), Al and Fe atoms in the *c*-axis chain are arranged in an ordered manner to form another low-temperature phase (η″) [23]. Those authors believed that the η″ phase has an incommensurately modulated crystal structure of long periodicity along the orthorhombic *c*-axis [23]. However, neither the occupation behavior of the Fe and Al atoms in the *c*-axis chain (i.e. their fractions at each of the Al2 and Al3 sites) nor the origin of the incommensurability/modulation was clarified. It is of particular interest to know the preferential sites for Fe/Al atoms and the manner of their ordered arrangement in the *c*-axis chain (if any), in view of the established principle for Al atoms in the *c*-axis chain in the η′ phase on the Al-rich side of the solubility range [24].

In the present study, we investigate the crystal structure of the η″ phase with two different compositions, by using single-crystal synchrotron X-ray diffraction (SXRD) and electron diffraction combined with atomic-resolution scanning transmission electron microscopy (STEM), as we have successfully applied to the crystal structure refinement of a series of intermetallics of the Fe-Zn system formed on galvanized/galvannealed steels [25–31].

## 2. Experimental procedures

Ingots with two different nominal compositions, Al-30.0 and 31.0 at.%Fe, were prepared by arc-melting Fe and Al (4N purity) under an Ar gas flow. Mass loss after the arc-melting was less than 0.1%. The solidified ingots encapsulated in a quartz ampoule with Ar gas were heat-treated under two different conditions: (i) at 900°C for 1 day and then at 300°C for 28 days followed by iced-water quenching, and (ii) at 1000°C for 1 day followed by iced-water quenching. The microstructures and chemical compositions of phases present in the specimens were examined by scanning electron microscopy (SEM) and transmission electron microscopy (TEM). Thin foils for TEM observations were prepared by twin-jet electro-polishing in a solution of perchloric acid and methanol (1:9 by volume) at −40°C and 10 V. Bright-field images and electron diffraction patterns were obtained with a JEOL JEM-2000FX TEM operated at 200 kV, while atomic-resolution images were obtained with a spherical-aberration-corrected JEOL JEM-ARM200F STEM operated at 200 kV. The probe convergence angle and the inner/outer detector angles for high-angle annular dark-field (HAADF) imaging were 22 and 90/370 mrad, respectively. Energy dispersive X-ray spectroscopy (EDS) was employed in atomic-resolution STEM observation. Simulations of STEM images and electron diffraction patterns were performed with the *WinHREM* software package [32] and *CaRIne* software [33], respectively. SXRD experiments were carried out at 300 K with a large cylindrical image-plate camera installed at BL02B1 of SPring-8 synchrotron radiation facility. A columnar specimen was machined from the η″ phase in the Al-31.0 at.%Fe alloy quenched from 300°C with a JEOL JIB-4000 focused ion beam (FIB) apparatus at an operating voltage of 30 kV. The size of the columnar specimen was approximately 18 μm in diameter and 20 μm in length. The crystal structure was solved by *SHELXT* [34] and refined by full-matrix least-squares techniques on *F*^2^ (*SHELXL-97*) [35] within the *WinGX* crystallographic software package [36,37].

## 3. Results

### 3.1. SEM and TEM observations

**Figure 2.**
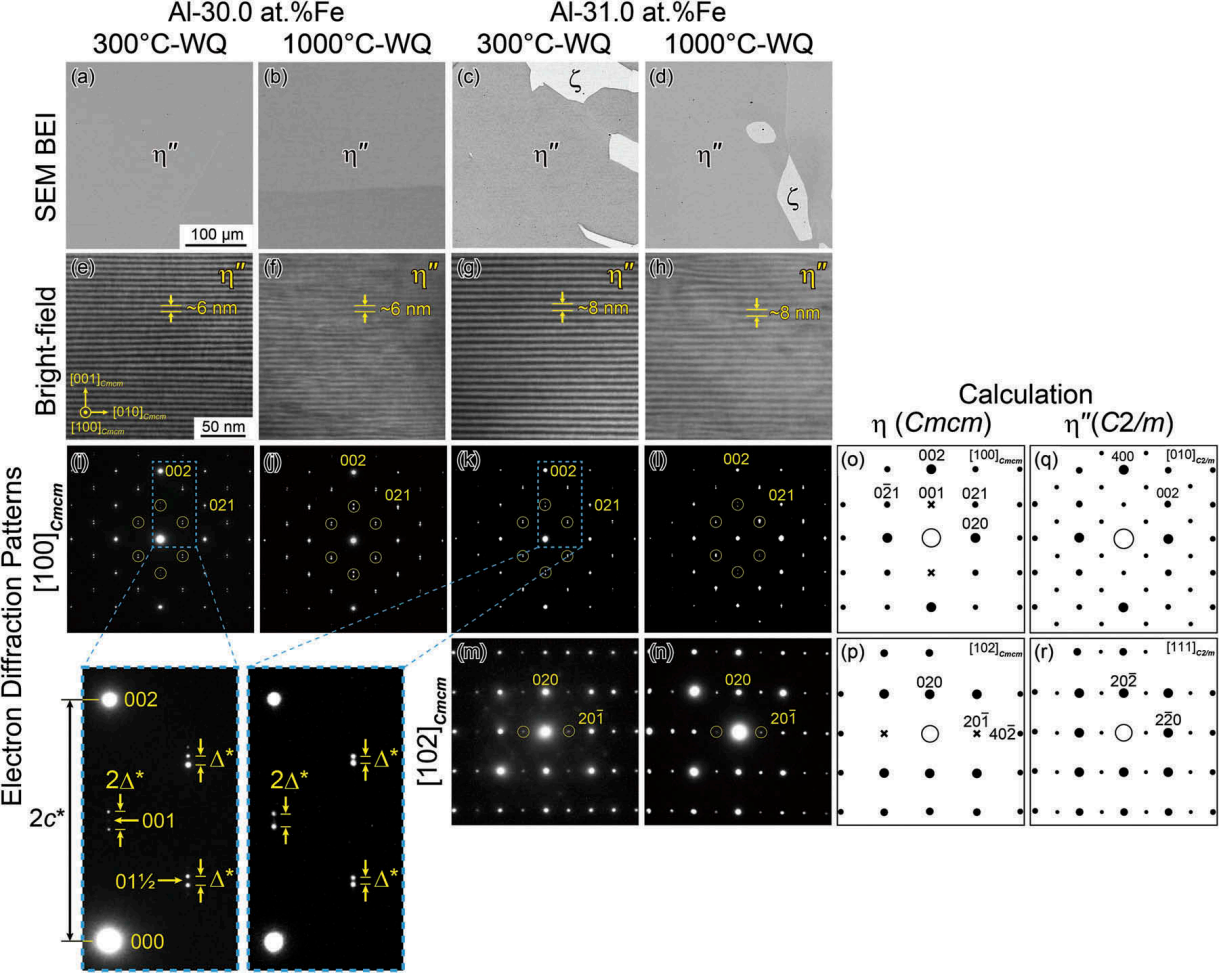
SEM BEIs of the microstructure observed in alloys with the nominal compositions of (a,b) Al-30.0 at.%Fe and (c,d) Al-31.0 at.%Fe at the two quenching temperatures. (e–h) TEM lattice fringe images and (i–n) SAED patterns with the [100]*_Cmcm_* incidence observed for the η″ phase in the alloys with the nominal compositions of (e,f,i,j) Al-30.0 at.%Fe and (g,h,k–n) Al-31.0 at.%Fe. Superlattice diffraction spots are indicated by yellow circles. Also shown are calculated SAED patterns of (o,p) the η-Fe_2_Al_5_ phase with the space group *Cmcm*, and (q,r) the motif structure of the low-temperature η″ phase with the space group *C*2/*m.*

Figure 2(a–d) shows SEM back-scattered electron images (BEIs) of microstructures in the two alloys with different nominal compositions quenched from different temperatures. While specimens with the Al-30.0 at.%Fe composition quenched from 300 and 1000°C both exhibit the η″ single-phase microstructure (Figure 2(a,b)), those with the Al-31.0 at.%Fe composition quenched from these two temperatures exhibit two-phase microstructures consisting of the major η″ phase (dark region) and the secondary ζ phase (bright region) (Figure 2(c,d)). This phase identification is based on electron diffraction analysis (not shown). Hence, the Fe-rich side boundary of the η″-phase solid solubility region is between Al-30.0 and 31.0 at.%Fe.

Figure 2(e–h) shows bright-field TEM images of the η″ phase with the [100]*_Cmcm_* incidence.^1^ All the four specimens exhibit lattice fringes parallel to the (001)*_Cmcm_* plane, with a spacing of approximately 6 and 8 nm for the Al-30.0 and 31.0 at.%Fe alloys, respectively. Figure 2(i–n) shows selected-area electron diffraction (SAED) patterns obtained from the η″ phase with the [100]*_Cmcm_* and [102]*_Cmcm_* incidences, respectively. All the SAED patterns contain superlattice diffraction spots in addition to those from the η phase with the space group *Cmcm* (Figure 2(o,p)). These superlattice diffraction spots appear at nearly the mid positions between the 000 and the 002, 021, and 0$\bar{2}$1 fundamental spots for the [100]*_Cmcm_* incidence, and between the 000 and the 20$\bar{1}$ fundamental (forbidden) spots for the [102]*_Cmcm_* incidence, respectively, as indicated by the yellow circles. Close inspection of the SAED patterns with the [100]*_Cmcm_* incidence (Figure 2(i–l)) indicates, however, that these superlattice diffraction spots are *not located exactly at these mid positions*, but rather split into two or more spots along the c* direction (as shown in the enlarged patterns in the bottom left of Figure 2). The split distance of these superlattice diffraction spots at the mid position between the 000 and the 002 fundamental spots (2Δ*) is twice that between the 000 and the 021 fundamental spots (Δ*). The split distance, 2Δ*, is approximately 1/15 and 1/19 the distances between the 000 and 002 fundamental spots (2c*) for the Al-30.0 and 31.0 at.%Fe alloys, respectively. As will be described in the following sections, the splitting of these superlattice reflections is due to structural/compositional modulation in the superlattice structure of the η″ phase. Specifically, the observed split distances indicate that the magnitude of the superlattice unit cell dimension of the η″ phase is respectively 15 and 19 times that of the *c*-axis of the parent orthorhombic unit cell of the η phase (0.42 184 nm). The spacing of the lattice fringes parallel to the (001)*_Cmcm_* plane (Figure 2(e–h)) is indeed fairly comparable to the magnitude of the superlattice periodicity (6.3 and 8.0 nm respectively for the Al-30.0 and 31.0 at.%Fe alloys) estimated from the split distance of the superlattice diffraction spots.

In the next two sections, we will describe the results of crystal structure refinement for the η″ phase in two steps: first determination of the motif structure without structural/compositional modulation (Section 3.2) and then determination of the long-period unit cell structure with structural/compositional modulation (Section 3.3).

### 3.2. Motif structure of the η″ phase without modulation

#### 3.2.1. Electron diffraction

**Figure 3.**
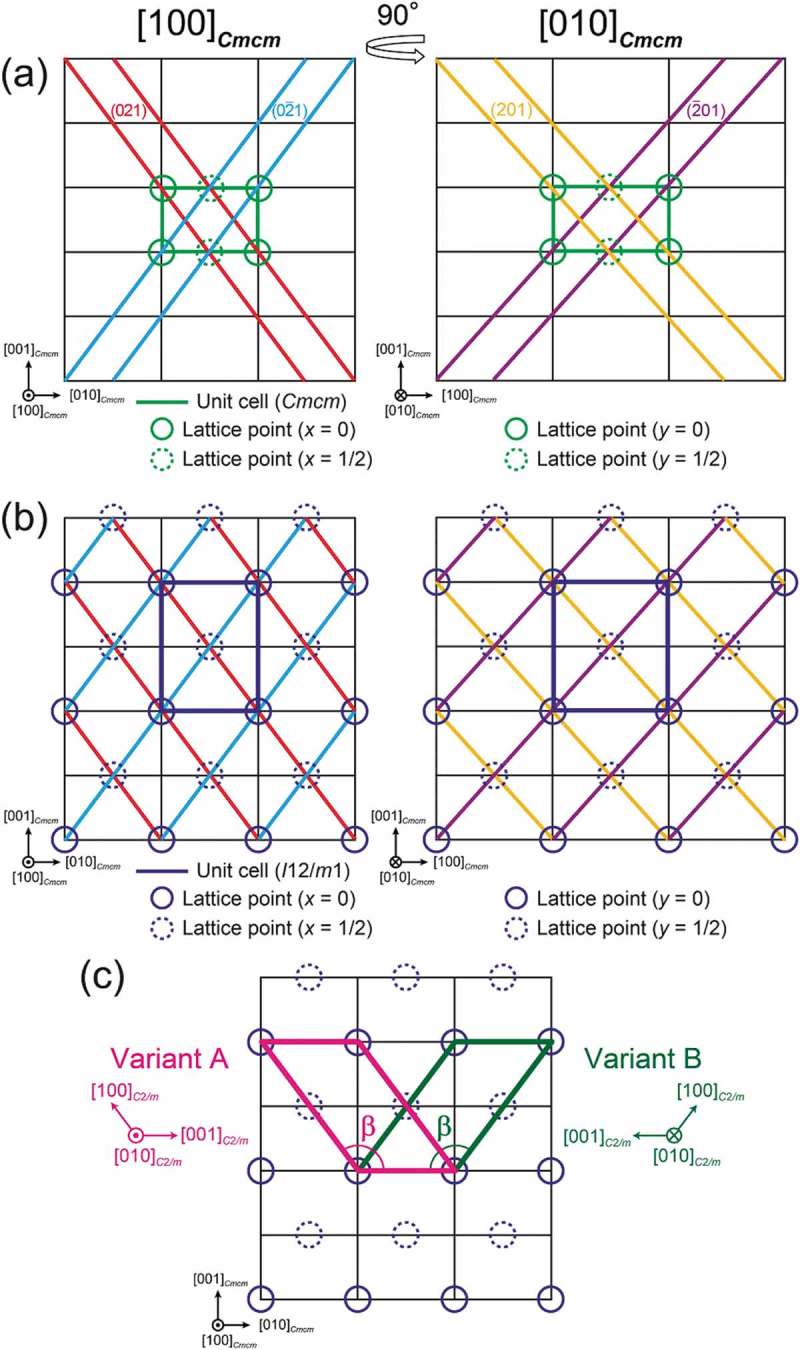
(a) Orthorhombic lattice of the η phase viewed along the [100]*_Cmcm_* and [010]*_Cmcm_* directions. The unit cell and the (0±21) and (±201) lattice planes are depicted in green, red/blue, and yellow/purple, respectively. (b) Body-centered orthorhombic lattice of the η″ phase. The unit cell and lattice points are depicted in dark blue lines and solid/dashed circles, respectively. (c) Unit cell choices for orientation variants A and B of the monoclinic η″ phase. The principal axes (*b*-axes) of A and B are parallel and antiparallel to the *b*-axis of the parent orthorhombic unit cell, respectively.

The unit cell dimension of the motif structure of the η″ phase without modulation could be deduced from the positions of superlattice diffraction spots observed in the SAED patterns (Figure 2(i–n)) by ignoring their splitting. Figure 3(a) shows the unit cell dimension (outlined in green) and interplanar spacing of (021), (0$\bar{2}$1), (201), and ($\bar{2}$01) lattice planes (indicated respectively with red, blue, yellow, and purple lines) of the η phase in the projection along the [100]*_Cmcm_* and [010]*_Cmcm_* incidences, which are orthogonal with each other. Since the superlattice diffraction spots are observed very near the mid positions between the 000 and 0±21 or ±201 fundamental spots (accompanied by splitting) (Figure 2(i–n)), interplanar distances for these four planes should be doubled in the η″ phase (Figure 3(b)). Intersections of these four lattice planes (dark blue circles) then form a body-centered orthorhombic lattice with a doubled *c*-axis lattice dimension. Therefore, the unit cell of the motif structure of the η″ phase is assumed to belong to the body-centered orthorhombic system possessing a unit cell volume twice that of the η phase. However, the atomic arrangement in the FeAl_2_ framework structure consisting of Fe and Al1 sites with full occupancy cannot be reproduced with any of the nine body-centered orthorhombic space groups (*Immm* and so on) listed in the *International Tables for Crystallography* [38]. Therefore, crystal symmetry of the motif structure in the η″ phase should be lower than body-centered orthorhombic (i.e. either monoclinic or triclinic). After symmetry considerations, we found it to be the base-centered monoclinic space group *C*2*/m*, which is equivalent to the ‘quasi’ body-centered orthorhombic space group *I*12/*m*1 (*β *≈ 90°) listed in the *International Tables for Crystallography* [38]. We have also confirmed that the base-centered space group *C*2*/m* (*β *≈ 127°) has the highest symmetry among all possible space groups.

Since the number of symmetry operations of the space group *Cmcm* (16) is twice that of *C*2/*m* (8), the η″ phase with the space group *C*2*/m* should have two orientation variants if it is formed from the η phase through solid–solid phase transformation upon cooling. These two orientation variants (designated as A and B) are generated by taking the principal axis (*b*-axis) of the monoclinic unit cell parallel (A) and anti-parallel (B) to the *a*-axis of the parent orthorhombic unit cell (Figure 3(c)). Since these two orientation variants are symmetric with each other with respect to the (001)*_Cmcm_* mirror plane, they are twins.

The Miller index transformation from the η phase (*Cmcm*) to the η″ phase (*C*2/*m*) for orientation variants A and B is expressed as follows:

Directions: [*uvw*]*_Cmcm_* → [*UVW*]*_C_*_2/*m*_

(1) $$\;\;\;\;\;\;\;\;\;\;\;\;\;\;\;\;\;\;\;\;\;\;\;\;\;\;\;\;\;\;\;\;\;\;\;\;\;\;\;\;\;U=w,\;\;\;V=2u,\;\;\text{\textbackslash{}break}W=2v+w\;\left(orientation\;variant\;A\right)$$

(2) $$\;\;\;\;\;\;\;\;\;\;\;\;\;\;\;\;\;\;\;\;\;\;\;\;\;\;\;\;\;\;\;\;\;\;\;\;\;\;\;\;\;U=w,\;\;\;V=-2u,\;\;\;\text{\textbackslash{}break}W=-2v+w\;\;\left(orientation\;variant\;B\right)$$

Planes: (*hkl*)*_Cmcm_* → (*HKL*)*_C_*_2/*m*_

(3) $$\;\;\;\;\;\;\;\;\;\;\;\;\;\;H=-k+2l,\;\;\;K=2h,\;\;\;\text{\textbackslash{}break}L=k\;\left(orientation\;variant\;A\right)$$

(4) $$\;\;\;\;\;\;\;\;\;\;\;\;\;\;\;\;\;\;\;H=k+2l,\;\;\;K=-2h,\;\;\;\text{\textbackslash{}break}L=-k\;\left(orientation\;variant\;B\right)$$

SAED patterns with the [010]*_C_*_2/*m*_ and [111]*_C_*_2/*m*_ incidences calculated for orientation variant A (corresponding respectively to the [100]*_Cmcm_* and [102]*_Cmcm_* incidences for the η phase) are shown in Figure 2(q,r), respectively. The experimental SAED patterns (Figure 2(i–n)) are well reproduced by the calculation (Figure 2(q,r)), except for the splitting of superlattice diffraction spots.

The approximate lattice parameters and atomic coordinates of the η″ phase with the space group *C*2*/m* (i.e. the motif structure without modulation) are derived based on those of the parent η phase [15] (left part of Table 1), and tabulated in the right part of Table 1. Each of the Fe, Al1, Al2, and Al3 sites in the η phase with the space group *Cmcm* splits into a few different Wyckoff sites in the η″ phase with the space group *C*2*/m*.

**Table 1.** Unit cell dimensions and atomic coordinates for (left) the parent η-Fe_2_Al_5_ phase [15], and (right) the motif structure in the η″ phase. The cell dimensions and atomic coordinates for the motif structure are simply derived from Ref [15]. The refined parameters are tabulated in Supplementary Table S2.

	η-Fe_2_Al_5_ (Burkhardt et al. [15])						η″ (Al-30.0 at.%Fe, not refined)					
Space group	*Cmcm*						*C*2/*m*					
*a* (nm)	0.76559						1.0599					
*b* (nm)	0.64154						0.7656					
*c* (nm)	0.42184						0.6415					
*β* (deg.)	−						127.25					
Cell volume (nm^3^)	0.20719						0.4144					
	Atom	Wyck.	Occ.	x	y	z	Atom	Wyck.	Occ.	x	y	z
	Fe1	4c	1	0	0.8277	1/4	Fe11	4i	1	0.125	0	0.453
							Fe12	4i	1	0.625	0	0.453
	Al1	8g	1	0.1880	0.1467	1/4	Al11	8j	1	0.125	0.312	0.271
							Al12	8j	1	0.125	0.188	0.772
	Al2	4b	0.32	0	1/2	0	Al21^a^	2a	1	0	0	0
							Al22	2d	0	0	1/2	1/2
							Al23	4i	0	0.750	0	0.250
	Al3	8f	0.24	1/2	0.034	0.830	Al31	4i	0	0.085	0	0.051
							Al32	4i	0	0.165	0	0.131
							Al33	4i	1	0.335	0	0.369
							Al34	4i	0	0.415	0	0.449

Reproduced with permission of the International Union of Crystallography.

^a^Al21 site is occupied by both Fe and Al atoms in the compositionally and structurally modulated structure of the η″ phase.

#### 3.2.2. STEM observation

**Figure 4.**
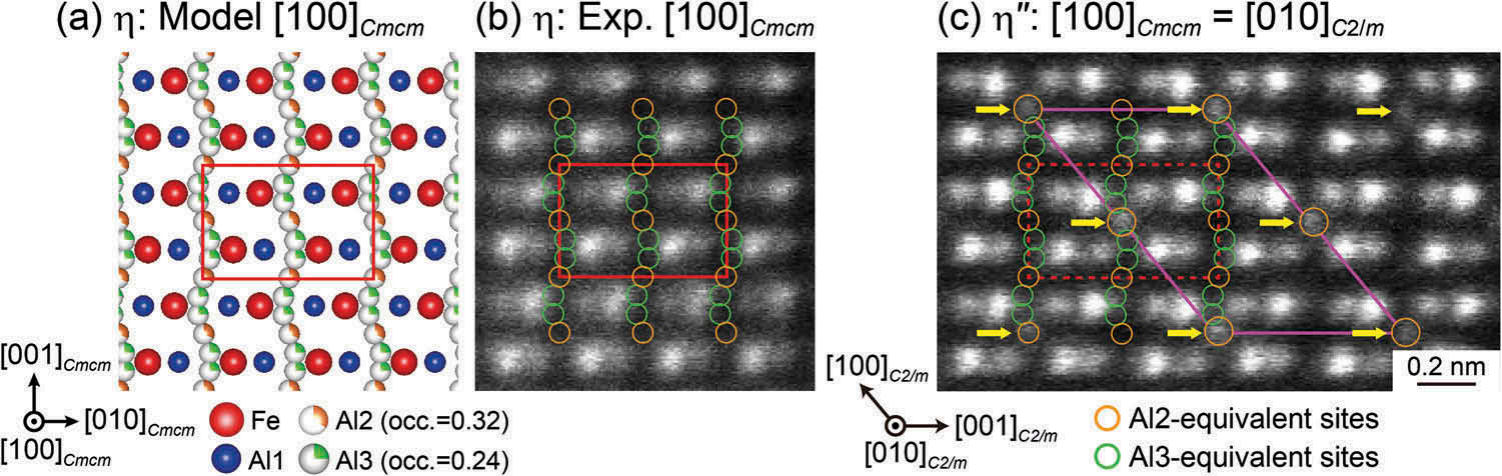
(a) Atomic arrangement of the η phase reported by Burkhardt et al. [15] projected along the [100]*_Cmcm_* orientation. HAADF-STEM images of the (b) η and (c) η″ phases taken along the [100]*_Cmcm_* direction, which corresponds to the [010]*_C_*_2/*m*_ direction for the monoclinic motif structure of the η″ phase.

Figure 4(b) shows an atomic-resolution HAADF-STEM image of the η phase with the [100]*_Cmcm_* incidence. The corresponding [100]*_Cmcm_* projection of the crystal structure for the η phase reported by Burkhardt et al. [15] is shown in Figure 4(a). While Fe atoms in the Fe sites and Al atoms in the Al1 sites with full occupancy forming the FeAl_2_ framework structure exhibit a well-defined asymmetric dumbbell pattern (Fe: brighter, Al1: darker), Al atoms at the Al2 and Al3 sites with partial occupancy in the *c*-axis chain do not exhibit any noticeable brightness contrast in the image. In the HAADF-STEM image of the η″ phase (Al-30.0 at.%Fe) with the equivalent [010]*_C2/m_* incidence (Figure 4(c)), on the other hand, bright spots are observed at positions corresponding to every fourth of the Al2 sites along the *c*-axis chain for the η phase, as indicated by yellow arrows. This is a clear indication that the Al (and Fe) atoms are arranged in the *c*-axis chain in an ordered manner, forming the monoclinic unit cell of the motif structure of the η″ phase with the space group *C*2*/m*, as outlined with magenta lines in Figure 4(c). The positions of these bright spots, indicated by yellow arrows, correspond to the Wyckoff 2*a* sites in the doubled monoclinic unit cell of the motif superlattice structure (right part of Table 1). As will be described later in Section 3.3.2, SXRD indicates that a part of the Al3 sites is also occupied by Al atoms in an ordered manner, although they are not visible in the HAADF-STEM image of Figure 4(c) due to their large atomic displacement parameters (Figure 6(c)).

### 3.3. Crystal structure of the η″ phase with modulation

#### 3.3.1. STEM observation

**Figure 5.**
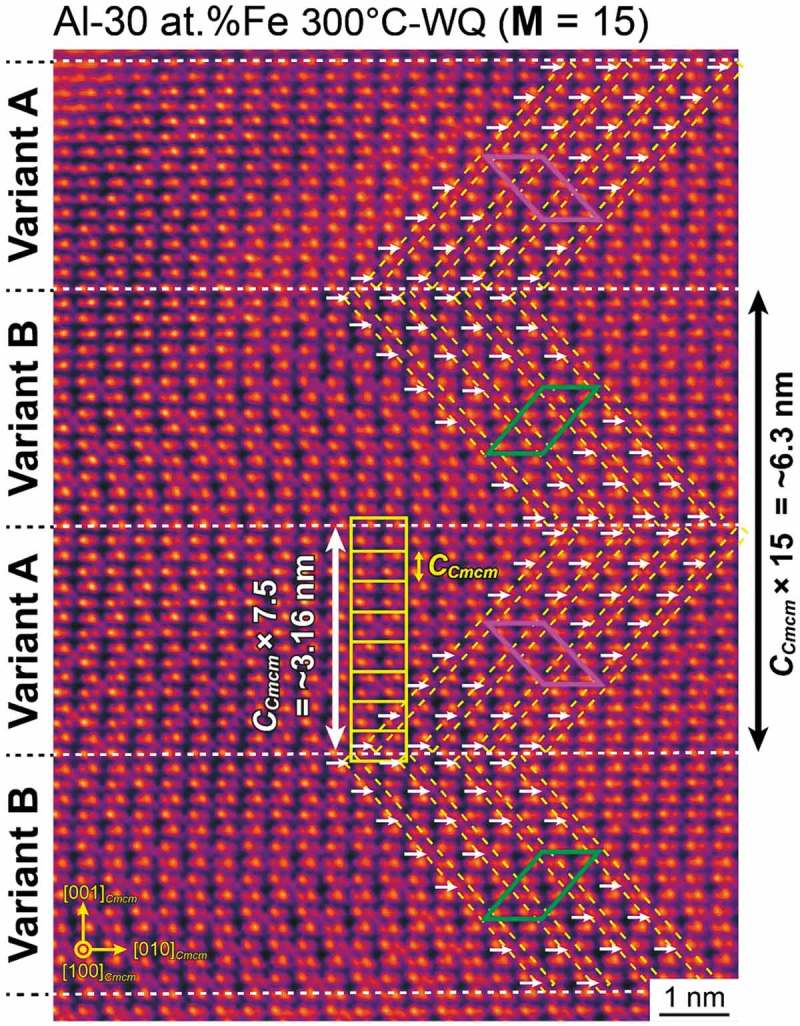
HAADF-STEM image of the η″ phase in the 30.0 at.%Fe alloy quenched from 300°C taken along the [100]*_Cmcm_* orientation. Regions with slightly bright contrast in the *c*-axis chain are highlighted by white arrows. The monoclinic unit cell of the motif structure of the η″ phase is indicated by magenta and green parallelograms.

Figure 5 shows an HAADF-STEM image of the Al-30.0 at.%Fe alloy quenched from 300°C taken along the [100]*_Cmcm_* direction. In addition to the asymmetric dumbbell pattern corresponding to Fe and Al atoms in the FeAl_2_ framework structure, there are clearly bright spots arranged in an ordered manner in the *c*-axis chain as highlighted by white arrows. These bright spots correspond to the Wyckoff 2*a* sites in the doubled monoclinic unit cell of the motif superlattice structure, as described in the previous section. Close inspection of Figure 5 indicates that there are two domains with different arrangements of these bright spots as outlined by yellow dotted rectangles (Supplementary Figure S1). These two domains are obviously symmetric with each other with respect to the (001)*_Cmcm_* mirror plane, and thus they are the twin variants described in Section 3.2.1. Surprisingly, the thickness of each twin variant is fairly constant when measured along the *c*-axis of the orthorhombic unit cell of the parent η phase, being 7.5 times the orthorhombic *c*-axis lattice constant (~3.16 nm) for this composition (Al-30.0 at.%Fe). Then, the total thickness of a pair of twin variants A and B is 15 times the *c*-axis lattice constant of the parent orthorhombic unit cell (M value^2^: 15) [39–41], amounting to approximately 6.3 nm, which is virtually identical to the spacing of lattice fringes (~6 nm) observed in the bright-field images of Figure. 2(e,f).

Thus, the crystal structure of the η″ phase can be described as consisting of twin (orientation) variants A and B alternately stacked along the *c*-axis of the orthorhombic unit cell of the parent η phase, with structural and compositional modulation at the twin boundary. Details of the structural/compositional modulation will be fully described in Section 4.2.

#### 3.3.2. Single-crystal SXRD

Structure refinement by single-crystal SXRD was made for the Al-31.0 at.%Fe alloy quenched from 300°C, and the details are given in Table 2. Many sharp reflections closely spaced in the range of reciprocal space (~0.12 nm^−1^) are observed, being consistent with a long periodicity of the η″ phase. But neither reflection broadening nor streaking was observed even when the image-plate was overexposed. Among the eight possible space groups suggested by the *SHELXT* software [34], the only centrosymmetric space group *Pmcn* (the 5th setting of *Pnma*) of the highest symmetry is assigned to the η″ phase. The *c*-axis lattice constant of the η″ phase is 8.01655 nm. This value is exactly 19 times that of the parent η phase (0.42184 nm) [15], and this is in good agreement with the spacing of lattice fringes (~8 nm) observed in Figure. 2(g,h) as well as the long periodicity estimated with the splitting distance of superlattice diffraction spots (Figure 2(l)). In further structure refinement, we employed the following procedures. At first, the Fe1−Fe19 and Al1−Al19 sites constituting the FeAl_2_ framework structure were assumed to be occupied exclusively by Fe and Al atoms, respectively, with full occupancy and isotropic displacement parameters. Meanwhile, the 14 different sites (Al21−Al25 and Al31−Al39) comprising the *c*-axis chain were assumed to be occupied exclusively by Al atoms. Then, mixed occupancies of Al and Fe atoms were allowed in each of the 14 different *c*-axis chain sites for the occupancy determination. Only one of the 14 chain sites was refined in each fitting procedure to avoid divergence of the fitting, and the site to be refined was changed one by one until the occupancies for all the *c*-axis chain sites were converged. As a result, we found that the Al31−Al39 sites are occupied almost exclusively by Al atoms (96 ± 7% on average) while the average Fe occupancy at the Al21−Al25 sites is as high as 27 ± 12% (Supplementary Table S1). Thus, the *c*-axis chain sites in the η″ phase are occupied by not only Al but also Fe atoms. This agrees with the result of crystal structure refinement for the η″ phase by Becker et al. [22,23], but is quite different from that of the η′ phase (Fe_3_Al_8_) reported in Ref [24] where the *c*-axis chain sites are exclusively occupied by Al atoms in an ordered manner. Finally, anisotropic displacement parameters for all the 52 sites were refined. Supplementary Table S1 gives the atomic coordinates and equivalent isotropic displacement parameters refined for the η″ phase in the present study.

**Table 2.** Summary of crystallographic data and structure refinement for the long-period ordered structure of the η″ phase in the Al-31.0 at.%Fe alloy quenched from 300°C.

Crystal data	
Refined chemical formula	Fe_20.74_Al_50.26_ (Al-29.2 at.%Fe)
Space group	*Pmcn* (the 5th setting of *Pnma*)
*Z*	4
Lattice constants, *a* (nm)	0.76603(2)
*b* (nm)	0.64285(1)
*c* (nm)	8.01655(24)
Cell volume (nm^3^)	3.9477
Density (calculated) (Mg/m^3^)	4.146
Formula weight	2464.11
Absorption coefficient (mm^−1^)	7.925
Crystal size (mm^3^)	0.018 × 0.018 × 0.020
*Data collection*	
Wavelength (nm)	0.04981
*θ* range for data collection (degree)	0.356 − 38.508
Index ranges	−19 ≤ *h* ≤ 16
	−16 ≤ *k* ≤ 16
	−99 ≤ *l* ≤ 173
Reflections collected	93,050
Independent reflections	26,355
Independent reflections (*I* > 2σ(*I*))	3042
Completeness to *θ*_max_	99.9%
*R*_int_	0.1137
*Refinement*	
Refinement method	Full-matrix least-squares on *F*^2^
Number of reflections used	26,355
Number of refined parameters	379
Goodness-of-fit on *F*^2^	0.977
*R*_1_ (*I* > 2σ(*I*))	0.0868
*wR*_2_ (*I* > 2σ(*I*))	0.2262
*R*_1_ (all data)	0.3270
*wR*_2_ (all data)	0.2795
Largest diffraction peak and hole (10^–3^ e⋅nm^−3^)	9.322/-5.552

Computer programs used: *WinGX* [36,37], *SHELXT* [34], and *SHELXL* embedded in *SHELX* [35].

**Figure 6.**
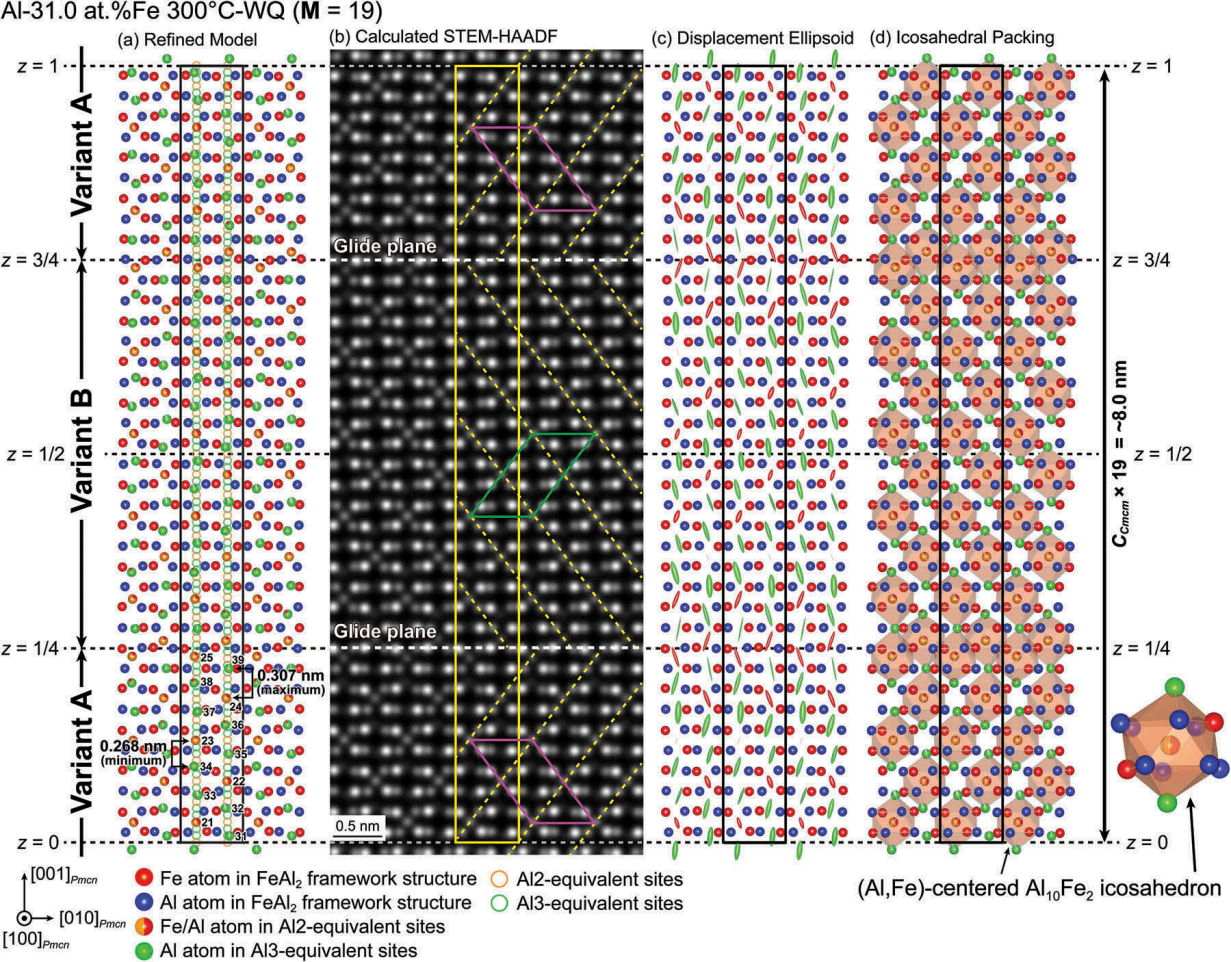
(a) [100]*_Cmcm_* projection of the refined long-period crystal structure of the η″ phase in the 31.0 at.%Fe alloy quenched from 300°C. The positions of the Al2- and Al3-equivalent sites (orange and green circles, respectively) are arranged in a straight line for simplicity. (b) HAADF-STEM image calculated based on the structural model refined by SXRD as shown in (a). The monoclinic unit cell of the motif structure of the η″ phase is indicated by magenta and green parallelograms. (c) Displacement ellipsoids (90% probability) of the Fe and Al atoms in the *c*-axis chain sites corresponding to the Al2 (red) and Al3 (green) sites in the parent η-phase structure. (d) Long-period crystal structure of the η″ phase described by the packing of distorted Al_10_Fe_2_ icosahedra whose center is occupied by Fe/Al atom (Al21–Al25 sites). The unit cell of the long-period crystal structure is indicated by black/yellow rectangles.

The refined atomic structure of the η″ phase in the Al-31.0 at.%Fe alloy is shown in Figure 6(a). This compound possesses a long-period superlattice structure based on the parent η-phase structure (space group *Cmcm*) with a peculiar ordered arrangement of Fe/Al atoms in the *c*-axis chain sites. While the orthorhombic unit cell of the η phase stacks along the *c*-axis to form a long-period superlattice unit cell of the η″ phase (M = 19 for Al-31 at.%Fe), almost every fourth of the *c*-axis chain sites is occupied in an ordered manner by Fe/Al or Al atoms, so that the unit cell consists of two twin-related domains alternately stacked along the orthorhombic *c*-axis. The top and bottom quarters of the unit cell correspond to the orientation variant A, while the central half of the unit cell corresponds to the orientation variant B in Figure 6(a). In other words, each of the twin domains is formed with the motif structure having the doubled unit cell dimension described in Section 3.2, and they comprise the unit cell of the η″ phase with the twin boundaries corresponding to the diagonal glide planes at *z* = 1/4 and 3/4. Importantly, it is the twin boundary that gives rise to the structural/compositional modulation that leads to the splitting of superlattice reflections. This is evident from the fact that while every fourth of the *c*-axis chain sites is ‘regularly’ occupied with the occupied-site sequence of …Al2/Al3/Al3/Al2/Al3/Al3… within the twin domains, this regularity is disturbed in the twin boundary region where the occupied-site sequence is …Al3/Al2/Al3/Al2/Al3…. The composition derived from the present structure refinement for the η″ phase is Al-29.2 at.%Fe, which is slightly lower in Fe content than the nominal alloy composition (Al-31.0 at.%Fe). Crystal structures of the η″ phase with different compositions can be constructed only by changing the number (M value) of the parent orthorhombic unit cells to be stacked along the orthorhombic *c*-axis (e.g. M = 15 for Al-30.0 at.%Fe), with identical atomic arrangements for the motif structure and the twin boundary.

Figure 6(b) shows an HAADF-STEM image calculated based on the refined structural model (Figure 6(a)) for the Al-31.0 at.%Fe alloy. It reproduces most features in the experimental HAADF-STEM image for the Al-30.0 at.%Fe alloy (Figure 5), except for the difference in M value. Al atoms at the Al31−Al39 sites are hardly visible in either the calculated or experimental images, because of the large atomic displacement of the corresponding sites (Figure 6(c)) [42,43]. Interestingly, the crystal structure of the η″ phase can also be described by the packing of distorted Al_10_Fe_2_ icosahedra whose center is preferentially occupied by an Fe atom (Al21−Al25 sites) as shown in Figure 6(d). This is very similar to the Fe-Zn intermetallic compounds (Γ-Fe_4_Zn_9_, Γ_1_-Fe_11_Zn_40_, δ_1p_-Fe_13_Zn_126_, and ζ-FeZn_13_), whose crystal structures are described by the packing of the common structural unit (Fe-centered Zn_12_ and/or Fe-centered (Zn,Fe)_12_ icosahedron) [25–31].

#### 3.3.3. EDS elemental mapping

**Figure 7.**
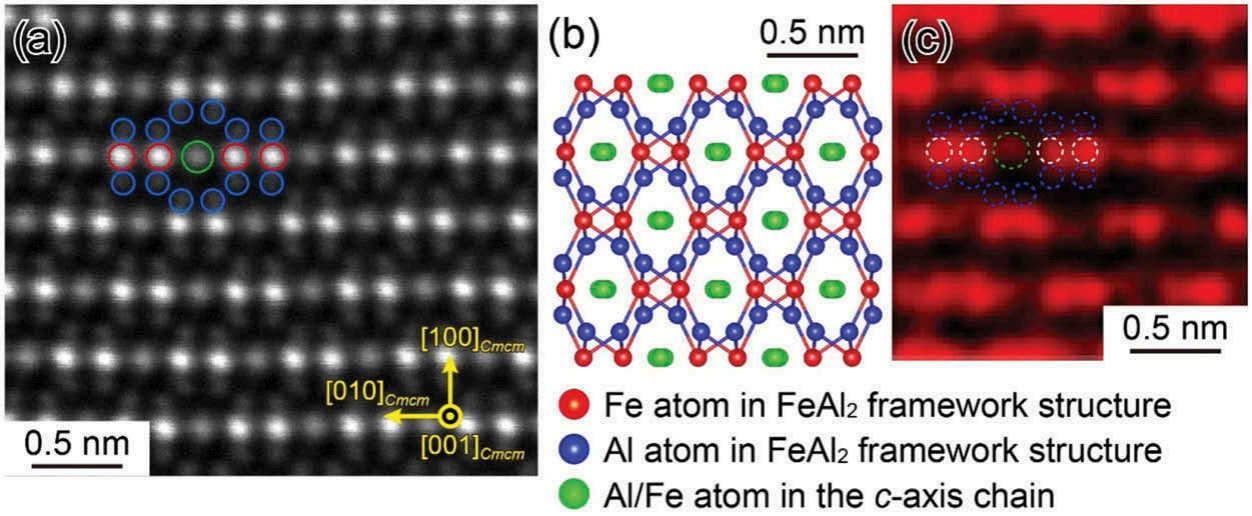
(a) HAADF-STEM image and (c) EDS Fe-elemental map of the η″ phase in the 31.0 at.%Fe alloy quenched from 300°C taken along the [001]*_Cmcm_* orientation. (b) Schematic illustration of the η″ structure viewed along the [001]*_Cmcm_* orientation.

Figure 7(a) shows an HAADF-STEM image of the η″ phase in the Al-31.0 at.%Fe alloy taken along the [001]*_Cmcm_* direction. In the projection along this direction, atomic columns forming the *c*-axis chain can clearly be observed without disturbance by other atoms, as seen in the schematic crystal structure of Figure 7(b). In addition to bright spots corresponding to Fe atoms (red circles) and Al atoms (blue circles) in the FeAl_2_ framework structure, those corresponding to the atomic columns of the *c*-axis chain (green circle) are clearly seen with the brightness between those of Fe and Al atoms in the FeAl_2_ framework structure. An Fe-elemental map obtained using atomic-resolution EDS is shown in Figure 7(c). Areas enriched with Fe are located at the *c*-axis chain sites, in addition to those at Fe atoms in the FeAl_2_ framework structure. This is a clear indication that the *c*-axis chain sites in the η″ phase are indeed occupied by not only Al but also Fe atoms.

## 4. Discussions

### 4.1. General description of crystal structures of the η″ phase

**Figure 8.**
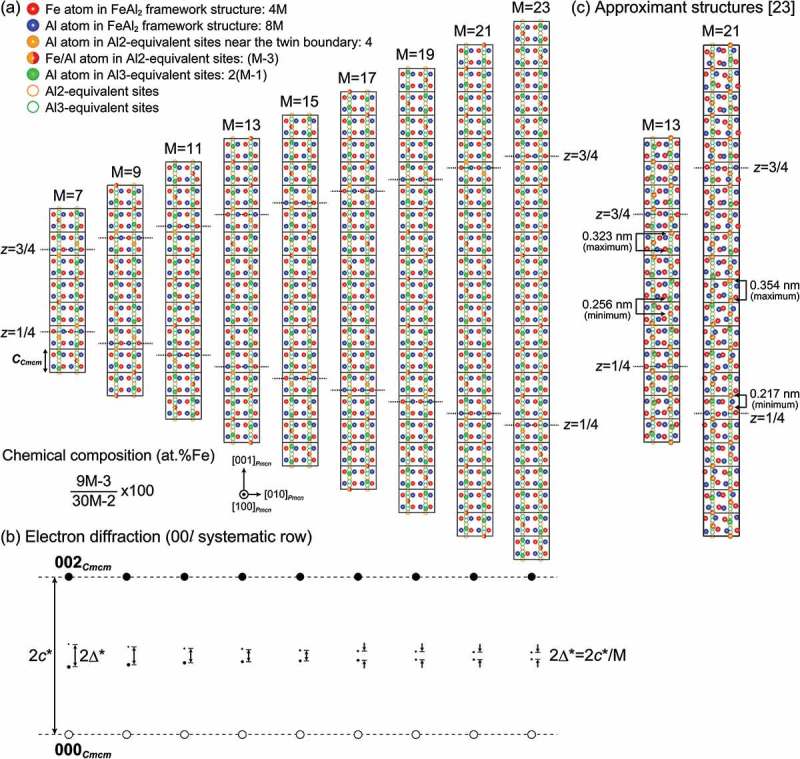
(a) Schematic illustration of the atomic arrangement in the commensurate long-period η″-phase structures for M = 7–23. For simplicity, it is assumed that the Al3-equivalent sites are occupied exclusively by Al atoms, while the Al2-equivalent sites are occupied evenly by Fe and Al atoms (50%:50%), except for the Al2-equivalent site closest to the twin boundaries that are occupied exclusively by Al atoms. The positions of the Al2- and Al3-equivalent sites (orange and green circles) are schematically arranged in a straight line for simplicity. (b) 00*l* systematic row in the SAED pattern with the [100]*_Cmcm_* incidence, calculated with the above atomic arrangement. (c) Approximant structures of the incommensurately modulated structures of the η″ phase with M = 13 (Al-29.3 at.%Fe) and M = 21 (Al-29.4 at.%Fe) as deduced by Becker and Leineweber [23].

In the present study, we determined that the η″ phase (the low-temperature phase of η-Fe_2_Al_5_ with Fe-rich side compositions) possesses an orthorhombic long-period superlattice structure (space group *Pmcn*), based on the parent orthorhombic unit cell (space group *Cmcm*) of the η phase. The superlattice structure consists of M unit cells of the parent orthorhombic lattice stacked along the orthorhombic *c*-axis, with a peculiar ordered arrangement of Fe/Al atoms in the *c*-axis chain sites. As a result, the unit cell of the η″ phase consists of two twin-related domains alternately stacked along the orthorhombic *c*-axis with two twin boundaries at *z* = 1/4 and 3/4, corresponding to the diagonal glide planes. Each of the twin domains is comprised of the motif structure with doubled unit cell dimension (Section 3.2). Based on the building principle described above, we can construct the crystal structures of the η″ phase with various compositions only by changing M among several values as shown in Figure 8(a). All these structures are commensurate with the modulation ‘regularly’ occurring at the interval of half the long periodicity. Indeed, the split distance (2Δ*) of superlattice diffraction spots in the SAED patterns with the [100]*_Cmcm_* incidence calculated for the structural models of Figure 8(a) is consistently 2c*/M, as shown in Figure 8(b). This clearly confirms the validity of our structure refinement result for the η″ phase (see also Supplementary Figure S2).

Becker and Leineweber [23] have recently reported that the η″ phase possesses *incommensurately* modulated structures using powder X-ray diffraction. The approximant structures^3^ they derived for the Al-29.3 and 29.4 at.%Fe alloys without taking the structural incommensurability into account are shown in Figure 8(c). These approximant structures are similar to the commensurate long-period structures determined in the present study in at least two aspects. First, the approximant/superlattice structures are based on the stacking of the parent orthorhombic unit cell of the η phase along the orthorhombic *c*-axis (the two structural models of Figure 8(c) can be expressed with M = 13 and 21). Second, the twin domains are stacked along the *c*-axis, with the diagonal glide planes at *z* = 1/4 and 3/4 being the twin boundaries. The key difference between the models of Becker and Leineweber [23] and ours (Figure 8(c) vs. 8(a)) is thus in how the Al/Fe atoms occupy the *c*-axis chain sites. The average distances of atoms located in the *c*-axis chain in these approximant structures (0.29 ± 0.02 and 0.29 ± 0.05 nm respectively for M = 13 and 21) are almost identical to that (0.29 ± 0.01 nm) in the commensurate long-period structure with M = 19 refined by SXRD in the present study (Figure 6(a)). This indicates that the atom packing density in the *c*-axis chain in these two structural models does not significantly differ from each other. However, their atomic arrangements along the *c*-axis chain sites are quite dissimilar. In our model, almost every fourth of the *c*-axis chain sites is ‘regularly’ occupied with the sequence of …Al2/Al3/Al3/Al2/Al3/Al3… (except for the twin-boundary regions), so that the motif structure is defined within twin domains for our commensurate structures (Figure 6(a) and 8(a)). However, such regularity in the occupied-site sequence is lost in the approximant structures deduced by Becker and Leineweber [23]. This is evident from the fact that the atomic distance in the *c*-axis chain in their approximant structures ranges widely, from 0.256 to 0.323 nm for M = 13 and from 0.217 to 0.354 nm for M = 21 (Figure 8(c) and Table 3). Clearly, the atomic arrangement within each of twin regions varies significantly depending on the M value, so that no motif structure can be defined in the approximant structures deduced by Becker and Leineweber [23]. We speculate that the contradiction between these two studies comes from the difference in the analyzed sample size. Becker and Leineweber [23] may have deduced the incommensurately modulated structures because their powder sample included various crystallites with different M values. This is evidenced by the significant broadening of superlattice diffraction spots, which they had noticed [23]. On the contrary, our STEM and SXRD experiments were performed on a very limited volume (~100 nm^3^ for STEM and ~0.005 mm^3^ for SXRD) compared to that of conventional powder X-ray diffraction (usually ~10 mm^3^). As a result, in both our STEM and SXRD analyses, the structure was homogeneous throughout the small volume of specimen, and as a result no broadening of superlattice diffraction spots was noticed (Section 3.3.2).

**Table 3.** Average, minimum, and maximum distances between Fe/Al atoms in the *c*-axis chain sites in the η″ phase.

M value	19^a^	13^b^	21^c^
Average (nm)	0.29 ± 0.01	0.29 ± 0.02	0.29 ± 0.05
Minimum (nm)	0.268	0.256	0.217
Maximum (nm)	0.307	0.323	0.354

^a^Al-31.0 at.%Fe alloy refined by SXRD (present study).

^b^Al-29.3 at.%Fe alloy refined by powder X-ray diffraction [23].

^c^Al-29.4 at.%Fe alloy refined by powder X-ray diffraction [23].

### 4.2. Composition dependence of crystal structures of the η″ phase

The unit cell of the η″ phase consists of two twin-related domains alternately stacked along the orthorhombic *c*-axis. Each domain is formed with the motif structure having a fixed composition (Fe_9_Al_21_ or Fe_3_Al_7_). Meanwhile, the twin boundary region has a different fixed composition (Fe_6_Al_16_ or Fe_3_Al_8_), which gives rise to structural and compositional modulation to cause the splitting of superlattice reflections. Since the chemical compositions of the motif structure and twin boundary region are different from each other, that of the η″ phase may vary with the M value (i.e. how frequently the twin boundary region appears along the *c*-axis stacking direction).

The numbers of atomic sites in the commensurate long-period structure with an arbitrary M value can be expressed as a function of M:

- Fe sites comprising the FeAl_2_ framework: 4M
- Al sites comprising the FeAl_2_ framework: 8M
- *c*-axis chain sites equivalent to Al2 sites in the parent orthorhombic structure: M + 1
- *c*-axis chain sites equivalent to Al3 sites in the parent orthorhombic structure: 2(M-1)

**Figure 9.**
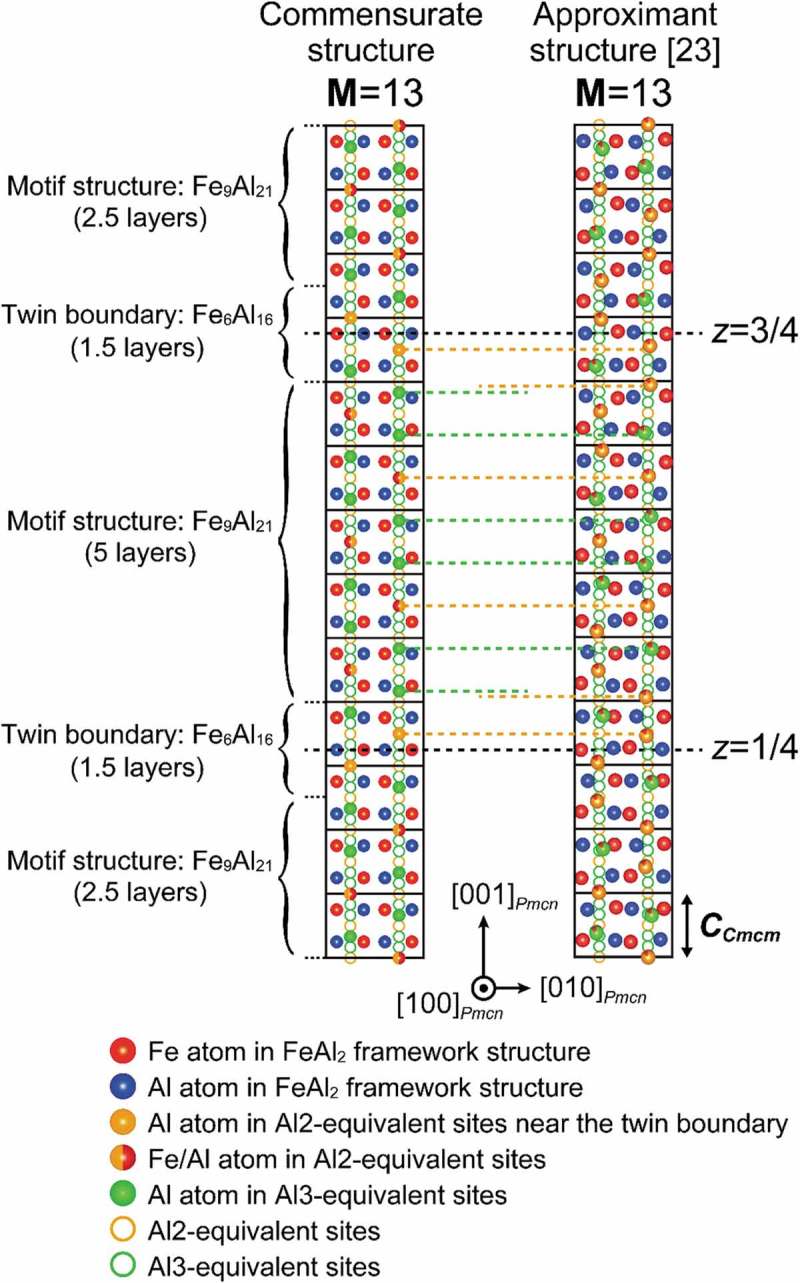
Comparison of the atomic arrangement in the *c*-axis chain sites, between the commensurate structure determined in the present study and the approximant structure deduced by Becker and Leineweber [23].

In total, there exist 15M-1 atomic sites per unit cell. As revealed by the SXRD analysis (Sections 3.3.2 and 4.1), the *c*-axis chain sites equivalent to Al3 sites in the parent orthorhombic structure (hereafter called Al3-equivalent sites) are occupied almost exclusively by Al atoms while those equivalent to Al2 sites (hereafter called Al2-equivalent sites) are occupied by both Fe and Al atoms. The exception is the Al25 site, closest to the twin boundary, where the occupancy of Fe toms is less significant compared with other Al2-equivalent sites (Figure 6(a)). Therefore, it is reasonable to assume that the Al3-equivalent sites are occupied exclusively by Al atoms while the Al2-equivalent sites are occupied evenly by Fe and Al atoms (50%:50%), except for the Al2-equivalent site closest to the twin boundary that are occupied exclusively by Al atoms. The numbers of Fe/Al atoms in each of the sites for several M values (from 7 to 23) are given in Table 4. As a result, the Fe content (chemical composition) in the compound is expressed as (9M-3)/(30M-2), increasing from 28.85 for M = 7 to 29.65 at.%Fe for M = 23 (bottom line in Table 4) and with the limit of 30.0 at.%Fe for M →∞. A similar conclusion can be drawn by considering the compositions of the motif structure and twin boundary with their respective layer thicknesses expressed with the M value. Since the layer thicknesses of the motif structure and the twin-boundary region are respectively M-3 times and three times the *c*-axis constant of the parent orthorhombic structure (Figure 9), the overall composition is expressed as (Fe_3_Al_7_)_3M-9_(Fe_3_Al_8_)_8_ = Fe_9M-3_Al_21M+1_. This tendency qualitatively agrees with our results: Al-30.0 at.%Fe for M = 15 and 31.0 at.%Fe for M = 19. Because the η″ phase always appears with twin-related domains (with fringe contrast in bright-field TEM images) regardless of the M value, they should be enriched in Al compared to the motif structure (Fe_3_Al_7_) by the incorporated amount of the twin boundary (Fe_3_Al_8_), and so the overall chemical composition is expressed as Fe_3_Al_7+*x*_.

**Table 4.** Numbers of Fe/Al atoms at each of the sites, and the chemical composition in the long-period η″-phase structures for several M values.

M value		7	9	11	13^b^	15^c^	17	19^d^	21^e^	23
FeAl_2_ framework sites, 12M	Fe, 4M	28	36	44	52	60	68	76	84	92
	Al, 8M	56	72	88	104	120	136	152	168	184
Al2-equivalent sites, M + 1	Al^a^, 4	4	4	4	4	4	4	4	4	4
	Fe (50%), (M-3)/2	2	3	4	5	6	7	8	9	10
	Al (50%), (M-3)/2	2	3	4	5	6	7	8	9	10
Al3-equivalent sites, 2(M-1)	Al (100%), 2(M-1)	12	16	20	24	28	32	36	40	44
Total Fe	(9M-3)/2	30	39	48	57	66	75	84	93	102
Total Al	(21M+1)/2	74	95	116	137	158	179	200	221	242
Total Fe + Al	15M-1	104	134	164	194	224	254	284	314	344
Chemical composition (at.%Fe)	(9M-3)/(30M-2)×100	28.85	29.10	29.27	29.38	29.46	29.53	29.58	29.62	29.65

^a^Nearest to the twin boundary.

^b^Al-29.3 at.%Fe alloy refined by powder X-ray diffraction (Becker and Leineweber [23]).

^c^Al-30.0 at.%Fe alloy observed by STEM (present study).

^d^Al-31.0 at.%Fe alloy refined by SXRD (present study).

^e^Al-29.4 at.%Fe alloy refined by powder X-ray diffraction (Becker and Leineweber [23]).

### 4.3. Comparison of the crystal structures of the η, η′, and η″ phases

**Figure 10.**
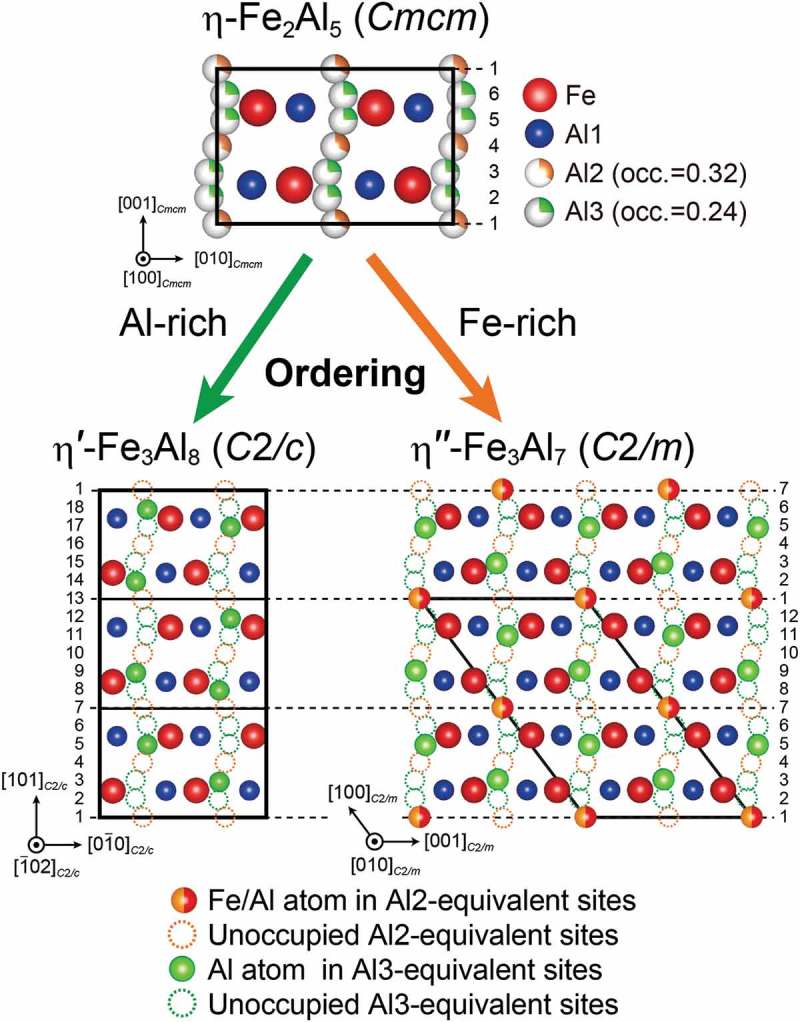
Structural relationships between the parent η-Fe_2_Al_5_, Al-rich low-temperature η′-Fe_3_Al_8_, and Fe-rich low-temperature η″-Fe_3_Al_7+*x*_ phases.

Although the crystal structures of the η′ and η″ phases are both superstructures based on the parent orthorhombic unit cell (space group *Cmcm*) of the η phase, the ordered manner in which Al/Fe atoms arrange in the *c*-axis chain sites is quite different from each other. Since the irregular atomic arrangement occurs in the twin boundary region, only the motif structure with the chemical formula of Fe_3_Al_7_ is considered for the η″ phase. In the η′ phase, one-third of the Al3-equivalent sites are occupied exclusively by Al atoms while all the Al2-equivalent sites are vacant (bottom left of Figure 10), giving rise to the tripled unit cell for the superlattice structure in the orthorhombic *c*-axis direction and the stoichiometric composition of Fe_3_Al_8_ [24]. Of the 18 Al2- and Al3-equivalent sites in the *c*-axis chain, the 5th, 9th, 14th, and 18th sites (i.e. two-ninths of the 18 sites) are occupied in a regular manner exclusively by Al atoms. In the η″ phase, on the other hand, one-fourth of the Al2-equivalent sites are occupied by both Fe and Al atoms, while one-fourth of the Al3-equivalent sites are occupied exclusively by Al atoms (bottom right of Figure 10), giving rise to the doubled superlattice structure and the stoichiometric composition of Fe_3_Al_7_. Of the 12 Al2- and Al3-equivalent sites in the *c*-axis chain, the 1st, 5th, and 9th sites (i.e. every fourth of the 12 sites) are occupied by both Fe and Al atoms. This confirms that Fe atoms also occupy the *c*-axis chain sites when the Fe content increases in the low-temperature phases (from η′ to η″ phases), as observed in the high-temperature η phase by Becker and Leineweber [23]. Interestingly, once the Fe atoms start to occupy the *c*-axis chain sites, the Fe atoms prefer to occupy the Al2-equivalent sites while the Al atoms change to prefer to the Al2-equivalent sites together with Fe atoms.

## 5. Conclusions

1. The η″ phase (the low-temperature phase of η-Fe_2_Al_5_ with compositions on the Fe-rich side of the solubility range) exhibits commensurate long-period superlattice structures (space group *Pmcn*) consisting of twin domains (orientation variants) alternately stacked along the long-periodicity axis parallel to the orthorhombic *c*-axis.
2. Each of the twin domains possesses a motif structure belonging to a base-centered monoclinic-ordered structure (space group *C*2/*m*), with a cell volume twice that of the parent orthorhombic unit cell of η-Fe_2_Al_5_ (space group *Cmcm*).
3. One-fourth of the *c*-axis chain sites corresponding to Al2- and Al3-sites in the η phase are respectively occupied by both Fe and Al atoms and exclusively by Al atoms in a regular manner, while this regularity is disturbed in the twin boundary region to give rise to structural/compositional modulation.
4. Because of the different chemical compositions in the motif structure and twin boundary region, the η″ phase with various compositions can be constructed only by changing the number (M) of the parent orthorhombic unit cells to be stacked along the orthorhombic *c*-axis, without changing the atomic arrangements for the motif structure or the twin boundary region.
5. The chemical formula of the η″ phase can be expressed as Fe_3_Al_7+*x*_ under a simple assumption about the occupancies for Al/Fe atoms in the *c*-axis chain sites.

## Notes

1. Subscript after the Miller index denotes the space group based on which the crystallographic directions and planes are indexed.

2. M denotes the number of the parent orthorhombic unit cells stacked in the superlattice unit cell of the η″ phase.

3. Approximant structures are commensurately approximated structures of the incommensurately modulated structures and derived by finding a small rational component close to the irrational component of the incommensurately modulated structures [23].
