# Supplementary material for: Crystal structure of η″-Fe3Al7+x determined by single-crystal synchrotron X-ray diffraction combined with scanning transmission electron microscopy
Source: Sci Technol Adv Mater. 2019 Jun 6;20(1):543–56. doi: 10.1080/14686996.2019.1613174 (PMC6566962; doi:10.1080/14686996.2019.1613174)
Supplement: Supplemental Material [file TSTA_A_1613174_SM5085.docx]

**Supplementary Information:**

**Crystal Structure of η’’-Fe_3_Al_7+_*_x_* determined by single-crystal synchrotron X-ray diffraction combined with scanning transmission electron microscopy**

Norihiko L. Okamoto^1,2,3^, Masaya Higashi^1^, and Haruyuki Inui^1,2^

^1^Department of Materials Science and Engineering, Kyoto University, Kyoto 606-8501, Japan

^2^Center for Elements Strategy Initiative for Structure Materials (ESISM), Kyoto University, Kyoto 606-8501, Japan

^3^(present address) Institute for Materials Research, Tohoku University, Sendai, 980-8577, Japan


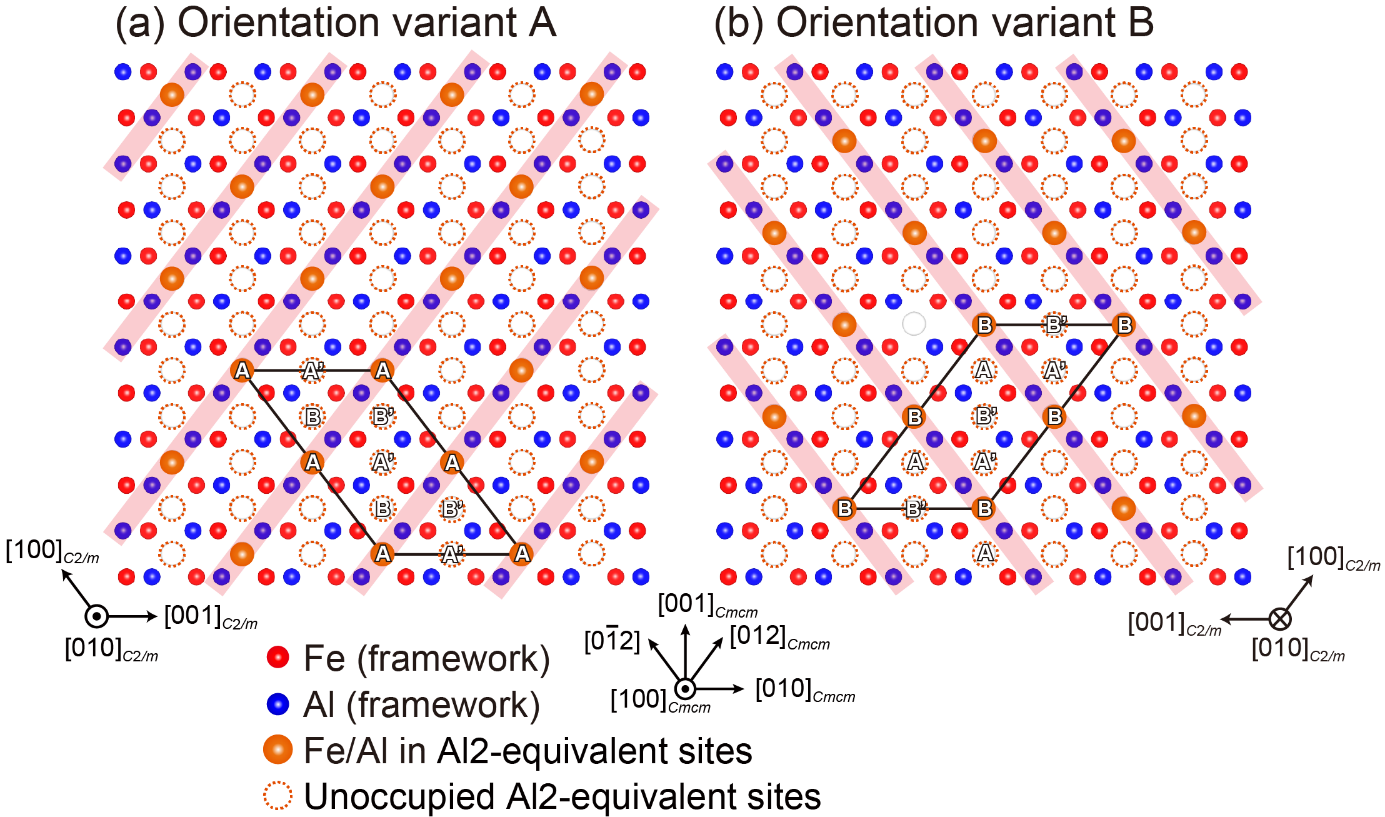


**Supplementary Figure S1.** Atomic arrangement of the twin domains (orientation variants) (a) A and (b) B viewed along the [100]*_Cmcm_* direction. The Fe/Al atoms occupy one-fourth of the Al2-equivalent sites (A, A’, B, and B’). The Fe/Al atoms in the *c*-axis chain (orange) and the Al atoms comprising the FeAl_2_ framework (blue) are arranged in an almost straight line along the oblique direction ([102]*_C_*_2/_*_m_* = <0±12>*_Cmcm_*) as highlighted by pale red strips. The Al3-equivalent sites in the parent η-phase structure are not shown in the figure for clarity.


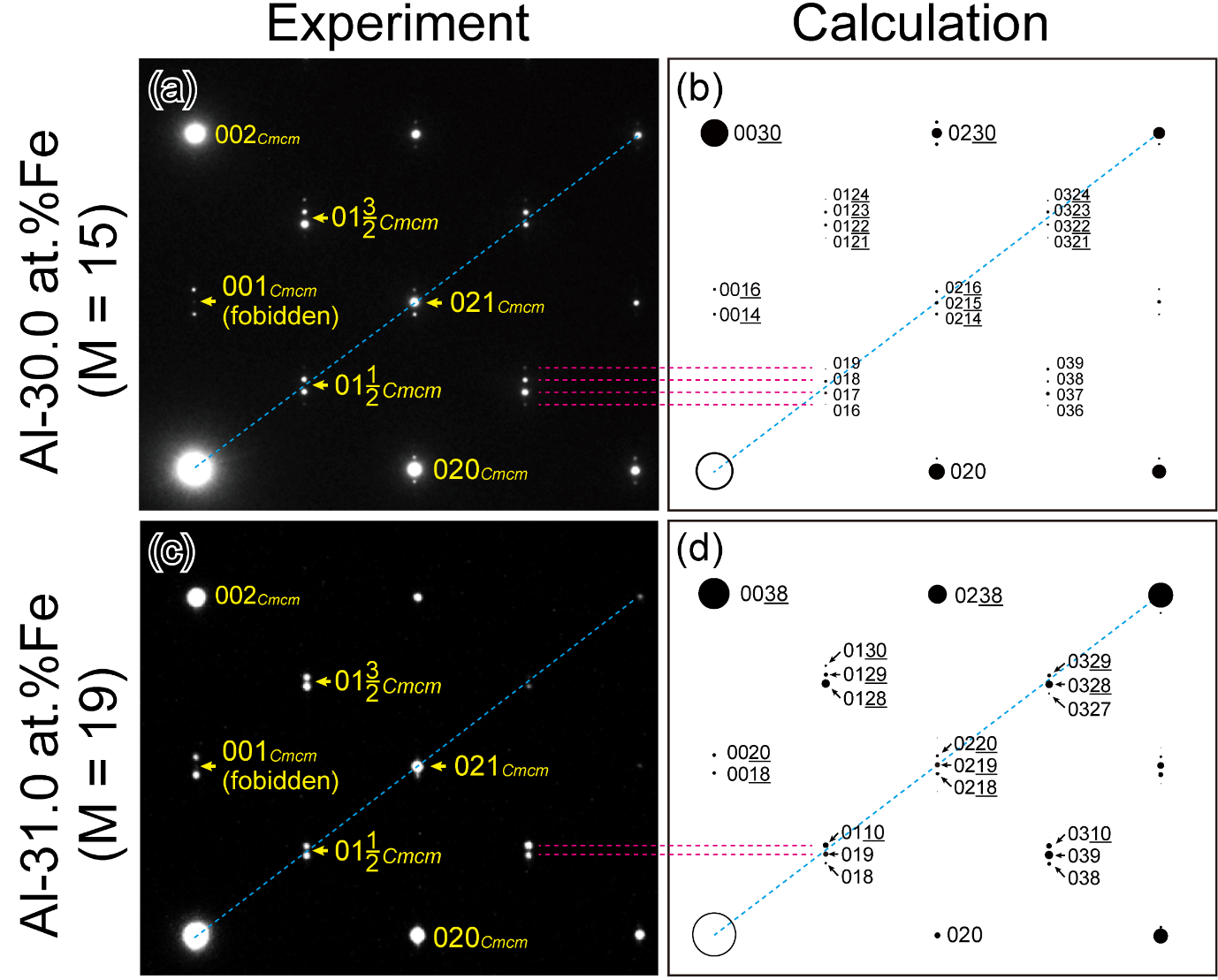


**Supplementary Figure S2.** (a,c) SAED patterns with the [100]*_Cmcm_* incidence experimentally observed for the (a) Al-30.0 at.%Fe and (c) Al-31.0 at.%Fe alloys. (b,d) SAED patterns with the [100]*_Cmcm_* incidence calculated with the atomic coordinates of the commensurate long-period structures for (b) M = 15 and (d) M = 19.

**Supplementary Table S1**. Atomic coordinates and equivalent isotropic displacement parameters *U*_eq_ for the long-period ordered structure of the η’’-phase compound refined in the present work. *U*_eq_ is defined as one-third of the trace of the orthogonalized *U^ij^* tensor.

| Atom | Wyck. | Occupancy (Al/Fe) | *x* | *y* | *z* | *U*_eq_ (pm^2^) |
| --- | --- | --- | --- | --- | --- | --- |
| Fe1 | 4*c* | 0/1 | 3/4 | 0.9171(2) | 0.01245(2) | 0.0080(2) |
| Fe2 | 4*c* | 0/1 | 1/4 | 0.4244(2) | 0.01400(2) | 0.0102(3) |
| Fe3 | 4*c* | 0/1 | 1/4 | 0.0715(2) | 0.03861(2) | 0.0096(3) |
| Fe4 | 4*c* | 0/1 | 3/4 | 0.5781(2) | 0.04026(2) | 0.0082(3) |
| Fe5 | 4*c* | 0/1 | 1/4 | 0.4135(2) | 0.06518(2) | 0.0082(3) |
| Fe6 | 4*c* | 0/1 | 3/4 | 0.9202(2) | 0.06658(2) | 0.0108(3) |
| Fe7 | 4*c* | 0/1 | 3/4 | 0.5676(2) | 0.09125(2) | 0.0094(3) |
| Fe8 | 4*c* | 0/1 | 1/4 | 0.0736(2) | 0.09291(2) | 0.0087(3) |
| Fe9 | 4*c* | 0/1 | 3/4 | 0.9101(2) | 0.11796(2) | 0.0078(2) |
| Fe10 | 4*c* | 0/1 | 1/4 | 0.4161(2) | 0.11905(2) | 0.0115(3) |
| Fe11 | 4*c* | 0/1 | 1/4 | 0.0648(2) | 0.14387(2) | 0.0087(3) |
| Fe12 | 4*c* | 0/1 | 3/4 | 0.5690(2) | 0.14556(2) | 0.0088(3) |
| Fe13 | 4*c* | 0/1 | 1/4 | 0.4072(2) | 0.17075(2) | 0.0079(2) |
| Fe14 | 4*c* | 0/1 | 3/4 | 0.9128(2) | 0.17147(2) | 0.0119(3) |
| Fe15 | 4*c* | 0/1 | 3/4 | 0.5634(2) | 0.19652(2) | 0.0083(3) |
| Fe16 | 4*c* | 0/1 | 1/4 | 0.0656(2) | 0.19821(2) | 0.0085(3) |
| Fe17 | 4*c* | 0/1 | 3/4 | 0.9059(2) | 0.22358(2) | 0.0082(2) |
| Fe18 | 4*c* | 0/1 | 1/4 | 0.4113(2) | 0.22381(2) | 0.0118(2) |
| Fe19 | 4*c* | 0/1 | 1/4 | 0.0636(2) | 0.24916(2) | 0.0084(3) |
| Al1 | 8*d* | 1/0 | 0.5697(4) | 0.5847(4) | 0.01358(3) | 0.0125(5) |
| Al2 | 8*d* | 1/0 | 0.0529(3) | 0.1192(4) | 0.01279(3) | 0.0118(5) |
| Al3 | 8*d* | 1/0 | 0.5691(4) | 0.9094(4) | 0.03904(3) | 0.0136(5) |
| Al4 | 8*d* | 1/0 | 0.0529(3) | 0.3770(4) | 0.03990(3) | 0.0117(4) |
| Al5 | 8*d* | 1/0 | 0.5523(4) | 0.6158(4) | 0.06548(3) | 0.0117(5) |
| Al6 | 8*d* | 1/0 | 0.0702(4) | 0.0797(4) | 0.06616(3) | 0.0130(5) |
| Al7 | 8*d* | 1/0 | 0.5550(4) | 0.8757(4) | 0.09255(3) | 0.0137(5) |
| Al8 | 8*d* | 1/0 | 0.0679(4) | 0.4035(4) | 0.09169(3) | 0.0154(5) |
| Al9 | 8*d* | 1/0 | 0.5703(3) | 0.5751(4) | 0.11873(3) | 0.0123(4) |
| Al10 | 8*d* | 1/0 | 0.0521(3) | 0.1121(3) | 0.11823(3) | 0.0110(4) |
| Al11 | 8*d* | 1/0 | 0.5656(4) | 0.8958(4) | 0.14429(3) | 0.0179(5) |
| Al12 | 8*d* | 1/0 | 0.0560(4) | 0.3751(4) | 0.14522(3) | 0.0139(5) |
| Al13 | 8*d* | 1/0 | 0.5524(3) | 0.6090(3) | 0.17091(3) | 0.0109(4) |
| Al14 | 8*d* | 1/0 | 0.0703(3) | 0.0712(3) | 0.17122(3) | 0.0126(4) |
| Al15 | 8*d* | 1/0 | 0.5585(4) | 0.8777(4) | 0.19786(3) | 0.0161(5) |
| Al16 | 8*d* | 1/0 | 0.4364(4) | 0.3885(4) | 0.19691(3) | 0.0181(5) |
| Al17 | 8*d* | 1/0 | 0.5704(3) | 0.5694(3) | 0.22375(3) | 0.0126(4) |
| Al18 | 8*d* | 1/0 | 0.4477(3) | 0.1073(3) | 0.22366(3) | 0.0107(4) |
| Al19 | 8*d* | 1/0 | 0.5613(4) | 0.8823(4) | 0.24951(3) | 0.0179(6) |
| Al21^†^ | 4*c* | 0.635(9)/0.365 | 3/4 | 0.2483(5) | 0.02645(4) | 0.0248(6) |
| Al22^†^ | 4*c* | 0.586(9)/0.414 | 1/4 | 0.7421(5) | 0.07931(4) | 0.0334(8) |
| Al23^†^ | 4*c* | 0.883(8)/0.117 | 3/4 | 0.2398(3) | 0.13182(3) | 0.0069(4) |
| Al24^†^ | 4*c* | 0.717(12)/0.283 | 1/4 | 0.7302(6) | 0.18629(6) | 0.0599(17) |
| Al25^†^ | 4*c* | 0.788(11)/0.212 | 3/4 | 0.2242(6) | 0.23990(8) | 0.069(2) |
| Al31^‡^ | 4*c* | 1/0 | 1/4 | 0.7822(8) | 0.00877(9) | 0.0651(19) |
| Al32^‡^ | 4*c* | 1/0 | 1/4 | 0.7191(8) | 0.04479(9) | 0.073(2) |
| Al33^‡^ | 4*c* | 1/0 | 3/4 | 0.2794(7) | 0.06124(9) | 0.0621(18) |
| Al34^‡^ | 4*c* | 0.986(11)/0.014 | 3/4 | 0.2157(8) | 0.09847(9) | 0.068(2) |
| Al35^‡^ | 4*c* | 1/0 | 1/4 | 0.7758(8) | 0.11443(9) | 0.063(2) |
| Al36^‡^ | 4*c* | 0.883(12)/0.117 | 1/4 | 0.7159(8) | 0.15176(10) | 0.090(3) |
| Al37^‡^ | 4*c* | 0.962(11)/0.038 | 3/4 | 0.2700(9) | 0.16894(11) | 0.086(3) |
| Al38^‡^ | 4*c* | 0.820(12)/0.180 | 3/4 | 0.2173(7) | 0.20572(8) | 0.088(3) |
| Al39^‡^ | 4*c* | 1/0 | 1/4 | 0.7719(6) | 0.22449(11) | 0.095(3) |

^†^Corresponding to the Al2 sites in the parent η structure. Center of (Al,Fe)_12_ icosahedra.

^‡^Corresponding to the Al3 sites in the parent η structure.

# **Structural refinement of the motif structure by first principles calculations**

The motif structure comprising each of the twin domains (A, B) in the long-period η’’-phase structure is refined by first principles calculations. The SXRD analysis on the long-period structure has suggested that the Wyckoff 2*a* sites (the Al2-equivalent sites) in the doubled superlattice structure with the space group *C*2/*m* are occupied by both Fe and Al atoms while the 4*i* sites (the Al3-equivalent sites) are occupied exclusively by Al atoms (right part of Table 1). Thus, a structural model in which the 2*a* sites are occupied by one Al and one Fe atoms are assumed (Supplementary Table S2)^^[[1]](#footnote-1)^^ so that its stoichiometry is expressed as Fe_3_Al_7_. The chemical composition of the structural model is Al-30.0 at.%Fe, which is in good agreement with the compositions of the generalized models for the commensurate long-period structures derived in the previous section (28.85−29.65 at.%Fe). The cell dimensions and atomic coordinates are refined by first-principles total-energy calculations based on density functional theory. We employed *VASP* code [1] based on the projector-augmented wave method within the generalized-gradient approximation of Perdew-Burke-Ernzerhof to the exchange-correlation functional. The plane-wave cutoff of 480 eV was used, and internal atomic positions, cell shape, and cell volume were relaxed until the residual forces became less than 10^-4^ eV/angstrom. The refined crystal parameters are tabulated in Supplementary Table S2. The cell volume of the refined structure is smaller by ~2% than that of the non-refined structure (right part of Table 1) but no significant anisotropic internal relaxation is noticed.

**References**

[1] G. Kresse, J. Furthmuller, Efficient iterative schemes for ab initio total-energy calculations using a plane-wave basis set, Phys. Rev. B 54 (1996) 11169-11186.

**Supplementary Table S2**. Unit cell dimensions and atomic coordinates for the motif structure of the η’’-Fe_3_Al_7+_*_x_* phase refined by first principles calculations. The base-centered translational symmetry is lost (*C*2/*m* 🡪 *P*2/*m*) due to the ordered arrangement of the Al and Fe atoms in the 1*a* and 1*e* sites, respectively, which correspond to the 2*a* sites of the space group *C*2/*m*.

| Space group | *P*2/*m* |  |  |  |  |
| --- | --- | --- | --- | --- | --- |
| *a* (nm) | 1.0609 |  |  |  |  |
| *b* (nm) | 0.7591 |  |  |  |  |
| *c* (nm) | 0.6369 |  |  |  |  |
| *β* (deg.) | 127.76 |  |  |  |  |
| *V* (nm^3^) | 0.4055 |  |  |  |  |
| Atom | Wyck. | Occ. | *x* | *y* | *z* |
| Fe11a | 2*m* | 1 | 0.1316 | 0 | 0.4596 |
| Fe11b | 2*n* | 1 | 0.6316 | 1/2 | 0.4668 |
| Fe12a | 2*m* | 1 | 0.6128 | 0 | 0.9328 |
| Fe12b | 2*n* | 1 | 0.1132 | 1/2 | 0.9407 |
| Al11a | 4*o* | 1 | 0.1308 | 0.3102 | 0.2640 |
| Al11b | 4*o* | 1 | 0.6271 | 0.2030 | 0.2468 |
| Al12a | 4*o* | 1 | 0.1209 | 0.1806 | 0.7817 |
| Al12b | 4*o* | 1 | 0.6218 | 0.3247 | 0.7957 |
| Al21a | 1*a* | 1 | 0 | 0 | 0 |
| Fe21b | 1*e* | 1 | 1/2 | 1/2 | 0 |
| Al33a | 2*m* | 1 | 0.3433 | 0 | 0.3877 |
| Al33b | 2*n* | 1 | 0.8148 | 1/2 | 0.3377 |

1. The base-centered translational symmetry is lost in the structural model for calculation due to the ordered arrangement of the Al and Fe atoms in the 1*a* and 1*e* sites, respectively, which correspond to the 2*a* sites of the space group *C*2/*m*. This ordering will not occur in the real crystal structure. [↑](#footnote-ref-1)
